# The Association of SARS-CoV-2 Infection and COVID-19 Vaccination With Sudden Death: An Explorative Review

**DOI:** 10.7759/cureus.89527

**Published:** 2025-08-07

**Authors:** Sakshitha Potluri, Nethra Chittiprol, Vamshi Varaganti, Vishnu AVR, Sabitha Vadakedath, Deepthi Arvapally, Chaitanya Vemulapalli, Gulam Saidunnisa Begum, Naveen Madamsetti, Venkataramana Kandi

**Affiliations:** 1 Medicine, Prathima Institute of Medical Sciences, Karimnagar, IND; 2 Health Sciences, Cypress Bay High School, Weston, USA; 3 Biochemistry, Prathima Institute of Medical Sciences, Karimnagar, IND; 4 Radiology, Emory Warner Robins /Houston Health Care, Warner Robins, USA; 5 Biochemistry, College of Medicine and Health Sciences, National University of Science and Technology, Sohar, OMN; 6 Inhalation, Proveris Scientific Corporation, Hudson, USA; 7 Clinical Microbiology, Prathima Institute of Medical Sciences, Karimnagar, IND

**Keywords:** cardiovascular system (cvs), central nervous system (cns), coronavirus disease-2019 (covid-19), covid-19 vaccination, sars-cov-2 infection, severe acute respiratory syndrome coronavirus-2 (sars-cov-2), sudden death (sd), sudden unnatural death (sd)

## Abstract

Since its discovery, the novel severe acute respiratory syndrome coronavirus 2 (SARS-CoV-2), the causative agent of coronavirus disease 2019 (COVID-19), has become the epicenter of public health concern. This was mainly attributed to the complexity of COVID-19 that resulted in variable disease progression with some developing asymptomatic infections, some suffering mild to moderate infections that resolved without the need for hospitalizations, and a few infected persons developing severe infections that required intensive care unit (ICU) admission and mechanical ventilation. The COVID-19 pandemic spread globally, affecting billions of people and killing millions. Most of the consequences were related to the novelty of the virus, poor understanding of its pathogenesis, and the lack of a specific antiviral drug and vaccine. The vaccines, although manufactured and made available to the public, were approved for emergency use before the completion of human clinical trials. Moreover, the continuous emergence of viruses following mutations resulted in the emergence of viral variants. This has led to doubts over the efficacy of vaccines. Vaccine inequity, represented by the disproportionate availability and distribution of vaccines among the rich and poor, concerns over long-term safety, and hesitancy, affected COVID-19 vaccination, thereby increasing the spread of SARS-CoV-2. Although the COVID-19 pandemic is no longer considered a public health emergency of international concern (PHEIC), the repercussions of the pandemic are still evident in the form of long COVID and post-COVID functional health status (PCFHS), wherein individuals who were previously infected continue to suffer organ dysfunction, primarily affecting the lungs and other organs of the body. During and after the pandemic, COVID-19 and probably vaccination were attributed to the death of many individuals, which were categorized as sudden death (SD) and sudden unnatural death (SUD). It is unclear if these deaths were a result of previous SARS-CoV-2 infection and prior COVID-19 vaccination or both. There are several instances of infected and recovered individuals who were healthy but suddenly developed complications and died. Through this explorative review, we aim to comprehend the role that SARS-CoV-2 infection and/or COVID-19 vaccination play in predisposing people to cardiovascular system (CVS) and central nervous system (CNS) disorders that can result in SD and SUD.

## Introduction and background

Severe acute respiratory syndrome coronavirus 2 (SARS-CoV-2) is the infectious agent that causes coronavirus disease 2019 (COVID-19), a highly contagious disease. COVID-19 has had a devastating effect, as more than six million people have died [1]. The presence of spike glycoproteins on the envelope gives coronaviruses (CoVs), which are positive-sense single-stranded RNA (+ssRNA) viruses, a crown-like look under an electron microscope [2]. The word "corona" means crown in Latin. In response to the thousands of deaths caused by COVID-19 and the global spread of SARS-CoV-2, WHO declared it a pandemic on March 12, 2020. Thus far, the pandemic has caused significant harm to the world's economies, increased poverty, and led to lost lives [3]. The first instance of SARS-CoV-2 infection in India was reported in Thrissur, Kerala, on January 30, 2020, among students returning from Wuhan, China [4]. As of March 27, 2022, India had the second-largest number of confirmed cases (43,019,453) and the third-largest number of COVID-19-related deaths (521,004). In response, the government implemented mass vaccination strategies to ensure immunity and protection for the entire country's population. A total of 1,830,285,290 doses had been administered to eligible individuals in India as of March 27, 2022. Of them, 1,003,924,602 received a single dose, representing 73% of the population, and 826,360,688 received all recommended vaccination doses, representing 60% of the population [5]. The first SARS-CoV-2 vaccine made in India, COVAXIN® (BBV152), was developed and manufactured by Hyderabad-based Bharat Biotech, a state-of-the-art biotechnology company, in association with the National Institute of Virology (NIV), Pune. The vaccine produced in India showed 64% effectiveness against symptomless patients, 93% against cases of severe SARS-CoV-2 infection, 78% against symptomatic cases, and 65% against the newly identified Delta variant [6]. Oxford University designed the Oxford-AstraZeneca COVID-19 vaccine (ChAdOx1 nCoV-19, codenamed AZD1222), which was marketed under the COVISHIELD™ brand and produced at the Serum Institute of India in Pune. The main vaccine utilized in India's mass vaccination campaign was COVISHIELD™, an AZD1222 made in India. In early January 2021, the Drug Controller General of India (DCGI) authorized it as the first vaccine for use in emergencies [7,8].

COVID-19 was noted to have variable clinical progression in different population groups; some infected individuals suffered asymptomatic infection, and a few developed symptomatic infections that required no hospitalization. Alternatively, some patients with COVID-19 needed hospitalization and intensive care admission, and treatment, including mechanical ventilation. The findings of a prior study showed that post-COVID-19 functional status (PCFS) is significantly restricted among recovered patients. A broad spectrum of physical and mental health issues that some individuals develop four weeks or more after contracting SARS-CoV-2 are referred to as PCFS. This includes people who initially had a moderate or asymptomatic acute illness. Comorbidities and immunization had a strong correlation with PCFS [9]. Although the vaccines against COVID-19 were made available very early during the pandemic, the primary reason why vaccines are not accepted in many countries globally is the side effects. However, a hurdle to immunization programs is the absence of accurate information from a reliable source, disinformation, and a lack of understanding and fear of negative effects. There have been numerous reports of side effects linked to the first or second doses of the booster vaccine. It appears that these related adverse events were either underreported or regarded as "usually rare." Interestingly, the Phase IV (human) clinical trials of these vaccines were taking place by default as the human population obtains these vaccines under the label of "emergency use” authorization by WHO [10]. Since its discovery, the SARS-CoV-2 virus has spread globally, making it difficult for the healthcare system to manage sick people and stop its spread. Despite global variations in mortality rates, COVID-19 and its effects on societal, cultural, political, and economic facets were universal. The early days of the pandemic were much more stressful because there was no specialized antiviral medication available, which led to higher rates of illness and mortality [11,12].

The cardiovascular, cerebrovascular, renal, blood and circulation, endocrine, and immunological systems are among the organ systems where cases of diverse presentations have been documented among COVID-19 patients. Acute coronary syndrome, myocarditis, pericarditis, thrombotic events, arrhythmias, hypertension, cardiac arrest, anemia, immunoglobulin nephropathy, tubulointestinal nephritis, acute disseminated encephalomyelitis (ADEM), rhabdomyolysis, and minimal change disease (MCD) were also identified as complications associated with SARS-CoV-2 infection [13]. A Thai study looked into the connection between sudden death (SD) or sudden unnatural death (SUD) and the COVID-19 vaccine. The study's findings suggested a possible genetic link between SCN5A polymorphisms and SD. Regardless of the type of vaccine, the number of doses, the existence of underlying conditions, or postvaccine fever, those with SCN5A variations may be linked to death within seven days of receiving the COVID-19 vaccine [14].

SD is defined as death that occurs naturally in a seemingly healthy person within hours of the onset of symptoms. Thus, SD refers to an unanticipated, generally unwitnessed, lethal event. A lot of these deaths happen when people are sleeping or at an unspecified time. The precise amount of time between the beginning of symptoms and death that must pass for an unexpected fatal event to be deemed sudden is up for debate. Any death that occurs within 24 hours of the onset of symptoms is considered SD, according to the WHO's most widely accepted criteria. Sudden cardiac death (SCD) is attributed to the majority of SDs. SCD results from heart failure (HF), which either stops beating or underperforms, resulting in hemodynamic collapse. Furthermore, it has been established that microbial infections by bacteria, viruses, fungi, and parasites can predispose to SD [15-17]. A previous study carried out among SD patients revealed that 41% of them had complained about cardiac symptoms like dyspnea, pedal edema, and chest pain [18].

An earlier randomized clinical trial study's findings showed that the COVID-19 vaccine was linked to more adverse event occurrences than a placebo [19]. Compared to conventional immunizations, mRNA vaccines behave more like pharmaceutical drugs. Since an mRNA vaccine is made up of a molecule called mRNA that must be transformed into an active agent called a protein or antigen to have pharmacological or immunological effects, it might be presumed to be a prodrug. Large and negatively charged, mRNA is unable to penetrate the cell membranes' anionic lipid bilayer. Lipid nanoparticles (LNPs), polyplexes, polymeric nanoparticles, peptides, and nanoemulsions are therefore necessary for the administration of these vaccines. Since these substances can probably affect how an mRNA vaccine behaves or how the host reacts to a vaccination, it is essential to evaluate their effects, taking pharmacodynamics (PD) properties like mechanism of action, pharmacokinetics (PK) including the absorption, distribution, metabolism, and excretion (ADME), and safety (adverse reactions) of the vaccine components both individually and in combination [20]. We examined the possible processes by which COVID-19 immunization and SARS-CoV-2 infection impact the cardiovascular and cerebrovascular systems in this review, and we comprehended the underlying mechanism that may potentially predispose individuals to SDs.

## Review

SARS-CoV-2 infection, COVID-19 vaccination, and cardiovascular consequences

COVID-19 is largely a respiratory illness; however, it has been linked to numerous cardiac problems. One of the most common consequences of the illness is cardiac injury. After COVID-19, myocarditis, arrhythmia, HF, and ischemic heart disease are among the long-term cardiac consequences. Research continuously demonstrates that the development of acute cardiac injury as a result of COVID-19 and underlying cardiovascular disease (CVD) in COVID-19 patients is linked to noticeably worse outcomes [21]. Similar to established CVD risk factors like obesity, diabetes, and hypertension, prior COVID-19 may raise the risk of several CVDs. Moreover, myocarditis, acute coronary syndrome, HF, thromboembolic consequences, and arrhythmia have all been linked to worse clinical outcomes and a higher risk of CVD in those who already have CVD. A chronic illness that persists for at least three months following a SARS-CoV-2 infection is known as the long COVID syndrome. Long COVID encompasses a broad spectrum of symptoms or presentations that may get better, become worse, or persist. Although some people who subsequently develop long COVID do not know when they were infected, the majority of people with long COVID start to exhibit symptoms days after first learning they have COVID-19. Multiple reinfections with SARS-CoV-2 are possible. Every time an individual contracts SARS-CoV-2, they run the risk of developing long COVID, which can predispose them to SCD [22,23].

Numerous and potentially fatal cardiovascular problems can result from COVID-19. Long COVID can have an impact on heart health. SARS-CoV-2 infection can cause microclotting [24]. A highly dynamic and changing field, long COVID has not yet been fully investigated. Key pathways in the pathophysiology of long COVID include an individual's immunological and inflammatory responses; pro-inflammatory molecules, including cytokines, may cause cardiac injury. Diabetes, arrhythmias, and HF are examples of new-onset metabolic and cardiovascular disorders that patients with Long COVID may experience [25].

**Figure 1:**
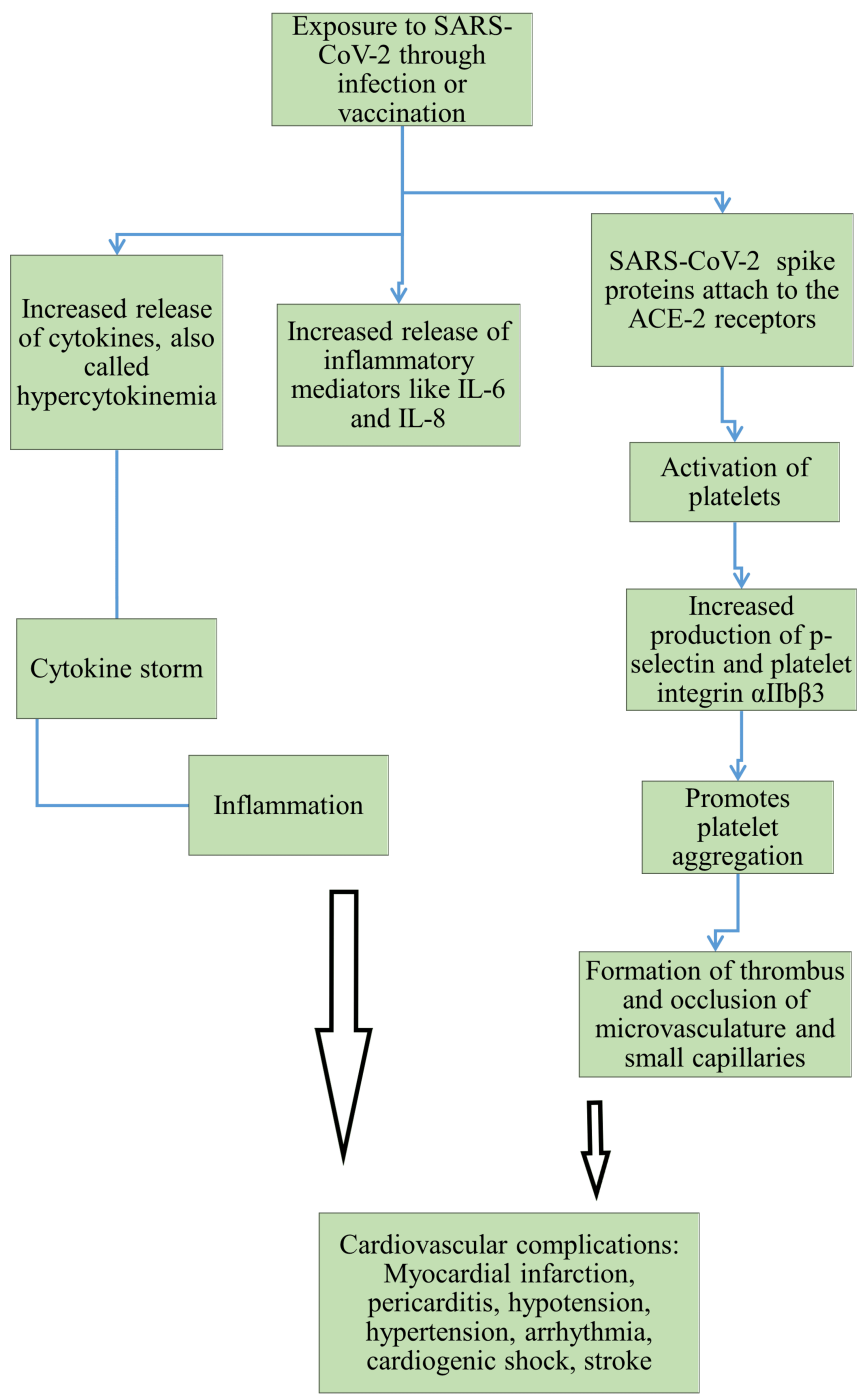
SARS-CoV-2 infection and COVID-19 vaccine mediated cardiovascular complications Image credit: The authors. This image has been synthesized from references [21-25,28,29]. SARS-CoV-2: severe acute respiratory syndrome coronavirus 2; COVID-19: coronavirus disease 2019; ACE-2: angiotensin-converting enzyme 2; IL: interleukin.

Myocardial ischemia, increased troponins, and acute HF with ventricular dysfunction are all possible outcomes of severe COVID-19, which may also increase the risk of intracardiac thrombus development, stroke, or systemic embolism [26]. The most common cardiac event following the COVID-19 immunization was myocarditis. The Moderna and Pfizer-BioNTech mRNA vaccines were the most common cause of all recorded cardiac events, including myocarditis, takotsubo cardiomyopathy (TTC), myocardial infarction (MI), and vaccine-induced thrombotic thrombocytopenia (VITT)/pulmonary embolism (PE). The incidence of myocarditis and TTC was decreased in cases with vector-based and/or inactivated vaccinations. COVID-19 vaccination with BNT162b2, mRNA-1273, and ChAdOx1-S was noted to be associated with SUD, referred to as out-of-hospital cardiac arrest (OHCA). Among more than 4.2 million persons aged between 5 and 50 years old who were vaccinated, 2,242 people experienced OHCA, and 38 succumbed to death within 30 days following vaccination [27-29]. A prior study that evaluated the contribution of COVID-19 and immunization to the development of SCDs found that young people had higher rates of SCD during the pandemic and following the implementation of the COVID-19 vaccine. A thorough autopsy revealed that the causes of SCD in young persons, including those who had SCD within 30 days after receiving the COVID-19 vaccine, were consistent with pre-pandemic causes, and there has been no discernible rise in the occurrence of myocarditis [29]. These conflicting observations indicate the necessity of improved understanding of the cardiovascular effects of SARS-CoV-2 infection and COVID-19 vaccination with SCDs. SARS-CoV-2 infections and COVID-19 vaccination can activate platelets. This could result in the activation of p-selectin and platelet integrin αIIbβ3. A vital receptor on the surface of platelets, platelet integrin αIIbβ3, is essential for both normal and anomalous blood clot formation. This can result in thrombosis and occlusion of blood vessels, potentially resulting in cardiovascular complications like myocardial infarction (Figure 1).

Mechanisms of SARS-CoV-2 and COVID-19 vaccination-related cardiovascular damage

Two significant pathogenic pathways of cardiovascular damage may be the direct harm caused by the SARS-CoV-2 infection and the indirect damage due to hyperimmune responses, including cytokine storm. A cytokine storm occurs when pro-inflammatory signaling molecules known as cytokines are released in excess and without control. Despite being a typical component of the body's immune response to infection, their abrupt and excessive release can result in multisystem organ failure and death. However, the exact pathophysiological processes of cardiovascular damage associated with COVID-19 are not entirely understood [30]. Heart rhythm problems are a serious concern, and there are warning signs in the literature regarding the possibility of post-vaccination malignant arrhythmias in certain susceptible patients, even though the risk-benefit ratio is unquestionably in favor of vaccination. It was observed that COVID vaccination with mRNA vaccines could affect cardiac electrophysiology and result in heart rhythm abnormalities [31]. The most frequent cardiac adverse event following the COVID-19 vaccine is myocarditis, which is also believed to be a possible cause of bradyarrhythmia. In patients who have received the COVID-19 vaccine, the following possible mechanisms may result in abnormal cardiac conduction and autonomic dysfunction: direct viral invasion through the production of spike (S) proteins and molecular mimicry; an increased inflammatory response; hypoxia; myocardial cell death; and eventual scarring or fibrosis. They are associated with a variety of disorders, such as bradyarrhythmia, ventricular arrhythmias, atrial tachyarrhythmias, SCD, and the common ailment known as myocarditis [32]. It is known that mRNA COVID-19 vaccination formulations contain trace amounts of leftover double-stranded RNA (dsRNA). It is commonly recognized that dsRNA can trigger immunological and inflammatory reactions. The unresolved cases of myocarditis may be caused by the presence of dsRNA in vaccination nanoparticles. TTC is an acute transitory left ventricular systolic failure that is differentially diagnosed from myocardial infarction. It has been suggested that TTC is caused by adrenergic stimulation, coronary vasospasm, microvascular dysfunction, inflammation, and changes in cellular metabolism. Acute emotional or physical stress raises blood levels of the hormone cortisol and catecholamines, as well as their bioavailability. These factors mediate a variety of pathways, including direct muscle cell damage, microvascular dysfunction, and pericardial coronary artery spasm, all of which are linked to TTC. Neural networks in limbic brain areas such as the hippocampus, prefrontal cortex, and amygdala are compromised in TTC patients under stress. Antibodies that target platelet factor 4 (PF4, also known as CXCL4) are the source of VITT, a potential reason for the thrombotic phenomena that occur after immunization [33].

**Figure 2:**
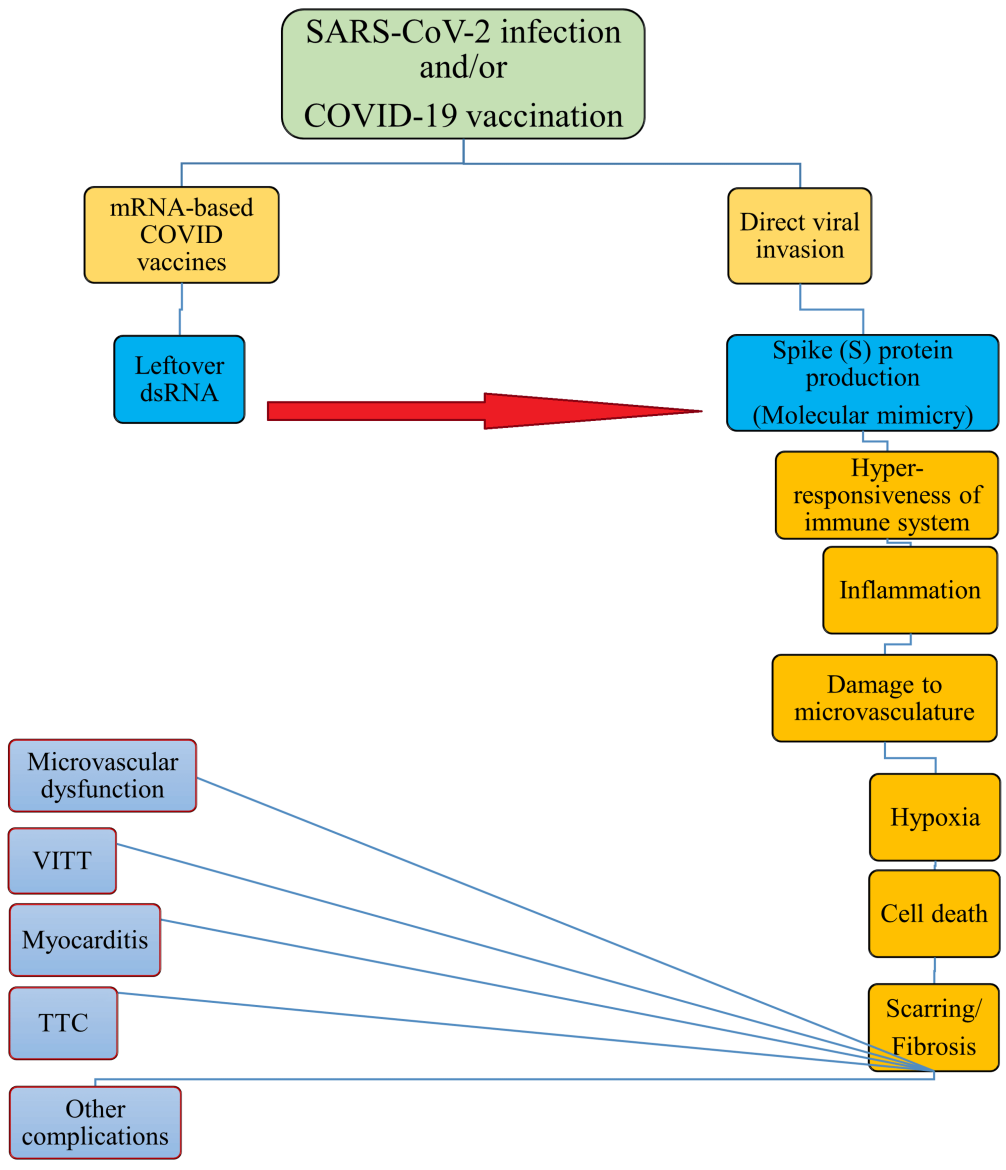
Mechanisms underlying SARS-CoV-2 infection and COVID-19 vaccination induced cardiovascular damage Image credit: The authors This image has been synthesized from references [26-29,31-33] SARS-CoV-2: severe acute respiratory syndrome coronavirus 2; COVID-19: coronavirus disease 2019; mRNA: messenger ribonucleic acid; dsRNA: double-stranded ribonucleic acid; VITT: vaccine-induced thrombotic thrombocytopenia; TTC: takotsubo cardiomyopathy

Coagulation abnormalities, respiratory problems, psychological disorders, and cardiac diseases were noted in a South Korean study that evaluated COVID-19 patients over 12 months following the onset of acute COVID-19 syndrome. After COVID-19, the risk of all-cause death increases for up to six months before sharply declining and declining within a year [34]. Acute cerebrovascular symptoms, like sudden speech confusion and limb paralysis; intracranial infection symptoms, like headache, epilepsy, and sleep disturbances; muscle damage symptoms, like limb soreness and weakness; and infrequently, symptoms like neuralgia and abnormal sensation are among the neurological symptoms that have been documented in COVID-19 patients thus far [35] (Figure 2).

SARS-CoV-2 infection, COVID-19 vaccination, and cerebrovascular consequences

Acute cerebrovascular disease has never been so strongly linked to an infection as the novel SARS-CoV-2 in the history of transmissible diseases. SARS-CoV-2 poses an unprecedented risk of ischemic and hemorrhagic stroke, even though there are documented links between the risk of stroke and other viral illnesses, such as influenza and the human immunodeficiency virus (HIV). The distinct correlation between SARS-CoV-2 and stroke can be explained by thrombus formation after cardiac dysfunction, increased thromboxane synthesis with related platelet activation, fast fibrinogen turnover, endothelial dysfunction, and inflammation. After infection, platelets are activated by the SARS-CoV-2 spike protein through platelet angiotensin-converting enzyme 2 (ACE-2) receptors, leading to increased production of P-selectin and platelet integrin αIIbβ3, which promotes platelet aggregation and degranulation.

Because the vascular endothelium expresses ACE-2 receptors on its surface, which allow viruses to enter endothelial cells and cause activation or disruption, it is also extremely vulnerable to viremia. Through inflammatory mediators like interleukin (IL)-6 and IL-8, which raise tissue factor expression and so trigger the extrinsic route, SARS-CoV-2 indirectly activates factor X [31].

**Figure 3:**
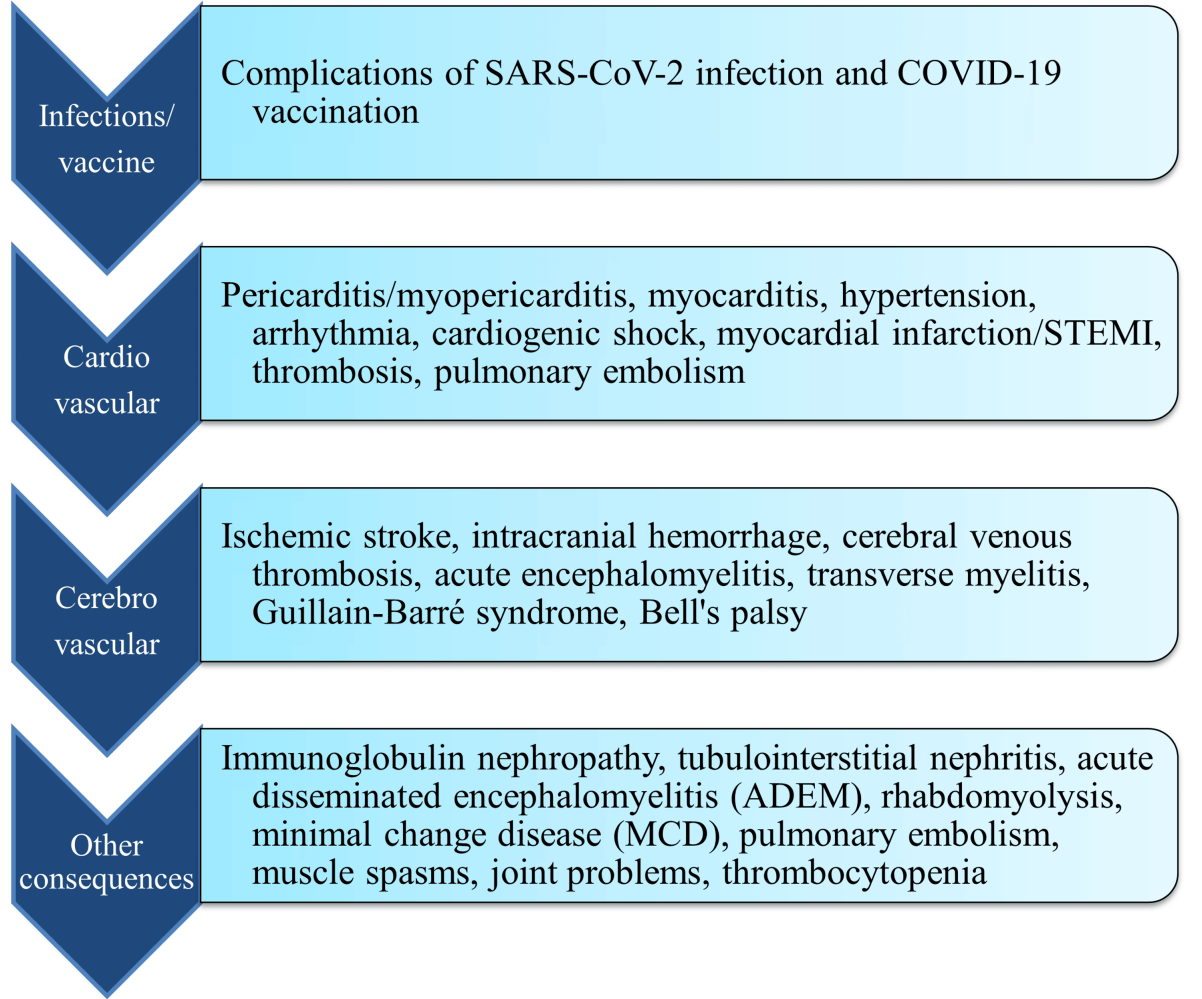
The consequences of SARS-CoV-2 infection and COVID-19 vaccination on central nervous system Image credit: The authors This image has been synthesized from references [21-25,28,29,35-38] SARS-CoV-2: severe acute respiratory syndrome coronavirus 2; COVID-19: coronavirus disease 2019; STEMI: ST elevation myocardial infarction

The first or second dose of the mRNA vaccines was associated with several cardiovascular adverse events, including pericarditis/myopericarditis, myocarditis, hypotension, hypertension, arrhythmia, cardiogenic shock, stroke, myocardial infarction/STEMI (ST elevation myocardial infarction), intracranial hemorrhage, thrombosis (deep vein thrombosis, cerebral venous thrombosis, arterial or venous thrombotic events, portal vein thrombosis, coronary thrombosis, microvascular small bowel thrombosis), and pulmonary embolism [36]. Weakness, numbness, headache, dizziness, imbalance, exhaustion, muscle spasms, joint discomfort, and restless leg syndrome are among the neurological adverse effects of the COVID-19 vaccine that are more likely to occur [37]. There have been numerous reports of serious neurological side effects from the COVID-19 vaccine, such as Bell's palsy, transverse myelitis, and encephalopathy. Following immunization, there have been reports of Guillain-Barré syndrome (GBS), cerebral venous thrombosis, and thrombocytopenia, as well as demyelinating disorders such as acute encephalomyelitis and transverse myelitis [38] (Figure 3).

Mechanisms of SARS-CoV-2 infection and COVID-19 vaccination-related cerebrovascular damage

**Figure 4:**
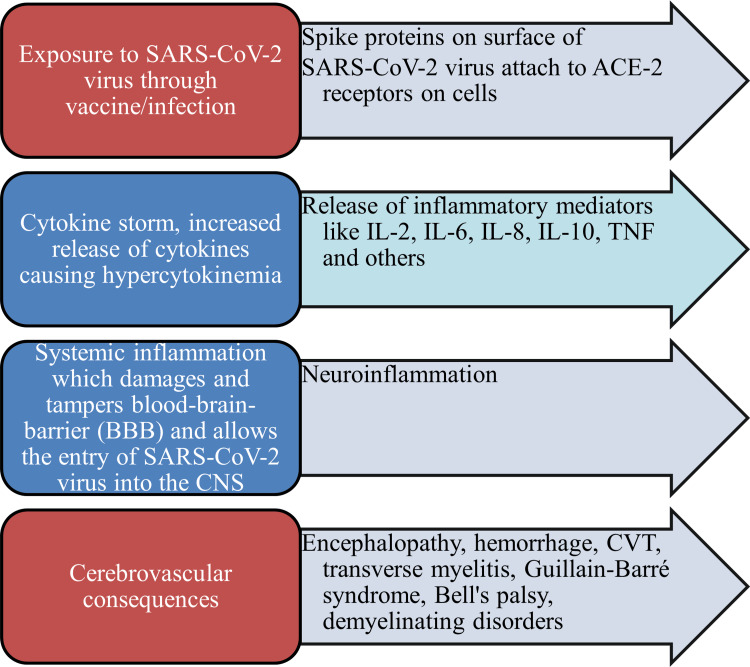
Mechanisms underlying SARS-CoV-2 infection and COVID-19 vaccination induced central nervous system damage Image credit: The authors This image has been synthesized from references [31, 35, 39-41] SARS-CoV-2: severe acute respiratory syndrome coronavirus 2; ACE-2: angiotensin-converting enzyme 2; IL: interleukin; CNS: central nervous system; TNF: tumor necrosis factor; CVT: cerebral venous thrombosis

Stroke and SARS-CoV-2 infection seem to be somewhat related. Activation of the proinflammatory cascade leading to coagulopathy, viral infection-induced platelet dysfunction or thrombocytopenia, and SARS-CoV-2-related vasculopathy with endothelial damage of small vessels are the three hypothesized mechanisms for the hemorrhagic stroke caused by the virus. Dysregulation of the crucial host cell receptor for SARS-CoV-2, the angiotensin-converting enzyme (ACE)-2-related physiological functions, endothelial cell damage, thrombo-inflammation, coagulopathy, and coagulation abnormalities associated with SARS-CoV-2 infection are some of the potential mechanisms for SARS-CoV-2-related ischemic stroke [39]. The novel SARS-CoV-2 is responsible for COVID-19, which can cause hypercytokinemia, also known as cytokine storm, which is a hyperinflammatory state marked by high cytokine levels and is typically seen in severe cases. Despite being largely a respiratory illness, COVID-19 has also been known to cause neurological problems that impact the central and peripheral nervous systems, potentially driven by cytokine storms and viral neuroinvasion. Clinical management using treatment targeting cytokine storm improved the understanding of the COVID-19-related cytokine storm and the neurological symptoms. In addition to monitoring recovered COVID-19 patients for post-infection neurological sequelae such as GBS, myositis, and Parkinsonism, which have been observed in previous coronavirus outbreaks, it will be crucial to analyze the presence of SARS-CoV-2 in cerebrospinal fluid (CSF), serum, and cytokine storm [40]. It has been noted that the proteins, receptors, and enzymes that help CoV-2 enter the central nervous system (CNS) through the blood-brain barrier (BBB) describe the consequences that follow, which can include neuroinflammation and mechanisms that disturb neurological symptoms [41] (Figure 4).

Influence of COVID-19 and vaccination on sudden deaths

The idea, definition, and clinical assignment of an SD and SUD are complicated and challenging, even with the development of several public health applications and useful attempts to define "SD" in the general situation. Historically, the primary risk factor for sudden fatality has been coronary artery disease. Other common risk factors include metabolic syndrome, diabetes, hypertension, left ventricular hypertrophy, chronic respiratory disease, mental illness, and substance misuse. These risk factors are consistent with pro-arrhythmic effects caused by ventricular fibrillation. For example, alcohol misuse can cause arrhythmia and untimely death [17]. Another scenario that could lead to SUDs is infection with various SARS-CoV-2 variants. SARS-CoV-2 and other viruses that are prevalent in the population may cause cardiac myopathies of different types, which are known to cause SUDs [28].

With the COVID-19 pandemic playing a major role, India's life expectancy at birth decreased moderately between 2017 and 2021. From roughly 49.7 years in the early 1970s to 70 years by 2016-2020, life expectancy at birth decreased to 69.8 years in the 2017-2021 period, according to the most recent health statistics. The tiny decline is seen as a key demographic indicator that shows how the pandemic has affected the country's population. It coincides with a dramatic rise in the number of fatalities reported nationwide. Over the preceding 20 years, the number of annual recorded deaths had been consistently between five and six million. However, in 2021, the second wave of COVID-19 caused an extraordinary spike in casualties, reaching over 10.2 million, a roughly 26% rise over the previous year. India's general mortality landscape was drastically altered by the magnitude of COVID-19 deaths and the resulting rising respiratory problems, which had a noticeable and long-lasting effect on national health statistics [42].

While age seems to play a major role in the course of the disease, gender, co-morbidities, and other genetic, physiological, and immunological traits of those who are afflicted have also been shown to have an impact on COVID-19 clinical outcomes [9,43-46]. This further demonstrates how intricate the COVID-19 pandemic is in comparison to earlier influenza pandemics.

COVID-19 vaccines in India

India started working on developing COVID-19 vaccinations during the initial pandemic wave. In April 2020, a special committee dedicated to vaccine research and development was formed. This research led to the initiation of clinical trials for vaccines such as Sputnik-V, ZyCoV-D, COVISHIELD™, and COVAXIN® by pharmaceutical companies such as Bharat Biotech, Zydus Cadila, Serum Institute of India (SII), and Dr. Reddy's Laboratories [47-49]. In January 2021, emergency use authorizations were granted for COVISHIELD™ and COVAXIN®, and subsequently, these two vaccines were given conditional market authorization in January 2022 [50]. After that, mRNA-1273 (Moderna) was authorized in late June, and Sputnik V was approved in April. Moreover, Zydus Cadila's ZyCoV-D and Johnson & Johnson's single-dose vaccine were approved in August [51, 52]. Since COVISHIELD™ and COVAXIN® were widely used throughout India, these are the primary subjects of our investigation. COVISHIELD™ and COVAXIN® played a key role in India's January 16, 2021, COVID-19 vaccination campaign. Over 2.2 billion doses had been given in India as of early March 2023, with COVISHIELD™ making up the majority of these doses [53]. Knowing how these vaccines are made, administered, and how they work is essential to comprehending their significance for public health.

*COVISHIELD*™

The SII-developed COVISHIELD™ is the Oxford-AstraZeneca vaccine's Indian equivalent. It is administered in two doses separated by 12 to 16 weeks. It has been shown that this extended schedule increases efficacy rates and strengthens the immune system [54]. ChAdOx1 nCoV-19, a modified chimpanzee adenovirus, is carried by COVISHIELD™ and acts as a vector to transfer the SARS-CoV-2 spike protein gene. It is made up of sodium dihydrogen phosphate dihydrate, sodium chloride, and disodium hydrogen phosphate dihydrate, which stabilize vaccination pH and function as buffers and preserve osmotic balance, respectively. Also, the vaccine contains polysorbate 80, which serves as an emulsifier to guarantee the vaccine's stability and homogeneity, and magnesium chloride hexahydrate, which stabilizes the viral vector and maintains its integrity. Water is utilized to inject the vaccine and serves as a solvent for dilution [55]. When the vaccine is administered, the ChAdOx1 vector enters human cells and causes them to produce the spike protein. Both humoral immunity (HI) and cell-mediated immunity (CMI) are boosted by this mechanism [56]. By inducing HI, the vaccine stimulates B cells to generate antibodies against the spike protein, neutralizing the virus. Moreover, vaccination triggers CMI, which results in CD4+T cells that target and eliminate contaminated cells while controlling immune responses.

*COVAXIN*®

COVAXIN® is an inactivated vaccine developed by Bharat Biotech and the Indian Council of Medical Research (ICMR). On January 2, 2021, it was authorized for emergency usage [57, 58]. It is administered in two doses, often separated by four to six weeks, similar to Covishield [59]. The inactivated SARS-CoV-2 virus in its dead form makes up Covaxin, which boosts immunity without producing a physical illness. Alum/aluminum hydroxide, an adjuvant used in the vaccine, augments the body's immunological response. The vaccine's Morris buffer stabilizes the vaccine and keeps its pH stable while it is being stored before being administered. The vaccine is administered intradermally after being diluted in water [55]. Inactivated viral particles are released into the body by COVAXIN®. Through the same mechanisms as COVISHIELD™, this exposure triggers an immune reaction. The HI generates neutralizing antibodies after identifying the inactivated virus. Any cells that could get infected with live virus particles are destroyed by CD4+T cells, which are activated by the CMI [59]. Research on the relationship between COVID-19 immunization and the prevalence of cardiovascular system (CVS) and CNS problems has become increasingly important, especially in the post-COVID pandemic age. Based on a thorough examination of a case of SD, our observational study identifies important mechanisms by which COVID-19 and the vaccine may have caused negative side effects, particularly neurological and cardiovascular issues.

Evidence 

In India, 47 tertiary care institutions were evaluated as part of a multicentric case-control study that looked at the causes and contributing factors of unexpected deaths in people between the ages of 18 and 45. Based on the findings of this investigation, the COVID-19 vaccination did not raise the risk of sudden, unexplained death among young adults in India. Certain lifestyle choices, a family history of SD, and previous COVID-19 hospitalization increased the chance of unexplained SD [60]. In contrast to this report, some researchers have contended that even though the study's authors found that hospitalization related to COVID-19 and family history were risk factors for SD among the cases, the fact that the controls survived for a comparable time (266.3 days) and that the cases died suddenly after 257.8 days does not necessarily rule out the role of the COVID-19 and vaccination. Similar to this, alcohol abuse and possibly the sort of vaccine that was given, which was not fully assessed, could have contributed to the abrupt deaths [61].

Based on autopsy results, a likely causative link between vaccination and the cause of death was evaluated. The average age of the deceased was 52.76 years, with 24 (70.59%) being men. Of them, 28 (82.36%), four (11.76%), and two (5.88%) received the AstraZeneca, Sinopharm, and Sinovac vaccinations, respectively. Ischemic heart disease was the leading cause of mortality. There was no link between the vaccine and any of the patients who passed away. Since there was no conclusive evidence in the study linking vaccination to the cause of death, deaths were attributed to natural causes [62]. However, to give the medical community a more thorough understanding of fatal COVID-19 vaccine-induced myocarditis, a prior study found many myocarditis-related deaths after COVID-19 immunization, which were verified by autopsies. According to the study's findings, there is a strong probability that COVID-19 vaccinations and myocarditis deaths are related. This might also be true in certain situations where a vaccinated individual has died suddenly and unexpectedly. Urgent research is needed for risk assessment and mitigation to lower the population's incidence of COVID-19 vaccine-induced myocarditis if the vaccinations are kept available for general use [63].

Counter-evidence

COVID-19 has been linked to a spike in SCD that cannot be explained in otherwise healthy, young people. We must consider the persistent trends in young people in both developed and developing nations, such as rising alcohol intake, smoking addiction, the sedentary lifestyle of the majority population, and psychological stress, all of which can harm the CVS. According to a cohort study of 7735 males in 24 locations in England, high alcohol use was found to increase the risk of SCD, and 117 (54%) sudden cardiac fatalities were reported [64]. Higher body mass (>28 kg/m^2^) and heavy drinking were found to be substantially linked to SCD (p<0.1) in current smokers [65]. For many years, stress has been linked to an increased risk of SCD. There may be both physical and psychological causes at play. Frequent psychological causes include financial losses, criminal offenses, involvement in traffic accidents (without major injuries), visits to the dentist, and the death or serious sickness of close family. Physical activity, sports, particularly swimming, sexual contact, alcohol and drug use, and physical pain are all examples of situations that can cause physical stress [66].

Despite its benefits for maintaining and growing muscle mass, the use of both legal and illegal anabolic-androgenic steroids (AAS) by young people is extremely cardiotoxic, leading to SCD in bodybuilders, athletes, and other young adults. Athletes' desire to have the ideal body and to boost their performance and self-esteem may account for the increased prevalence of AAS usage among them, particularly among non-professionals. Using AAS can lead to a decrease in body fat and an increase in muscular mass, strength, energy, and focus. The adverse effects of using AAS have the greatest impact on the CVS. According to reports, abusing AAS might encourage the growth of heart tissue, resulting in hypertrophic cardiomyopathy and apoptotic cell death. This process, which is linked to myocardial infarction, cardiomyopathy, ventricular remodeling, and SCD, can explain how AAS may cause cardiac mortality without atherosclerosis or coronary thrombosis [67].

Plan for further research and implementation

SARS-CoV-2 infection has been associated with cytokine storm, arterial and venous thromboembolisms, myocarditis, arrhythmias, and cardiovascular and cerebrovascular damage. Moreover, the viral dissemination into different organs of the body is facilitated by the availability of ACE-2 receptors on different cellular components. Long-COVID and PCFHS, leading to disability adjusted life years (DALYs), further confirm the long-term effects of the previous SARS-CoV-2 infection and COVID-19. These post-COVID complications have been noted to significantly affect the lives of people who suffered from COVID-19-related hospitalization and severe illness. Following COVID-19, an uncommon consequence that primarily affects some individuals is multisystem inflammatory syndrome (MIS). Unrelenting fever, rash, conjunctivitis, neurological symptoms, shock, gastrointestinal issues, and thrombocytopenia are common presentations. MIS may cause multi-organ damage and become a cause for SD and SUD. Myocarditis has been linked to the COVID-19 vaccine, and there have been worries regarding potential SCD from the vaccine. Some observations revealed deaths among those who got a dose of the mRNA COVID-19 vaccine within 100 days of the vaccination. There is an urgent need to study the long-term cardiac complications associated with COVID-19 vaccines.

A causal relationship between SARS-CoV-2 infection and COVID-19 vaccinations, and SD cannot be accepted or rejected, despite some evidence linking COVID-19 and immunization to SD, SUD, and SCD. Understanding this phenomenon requires additional human, animal, and experimental research. Improved knowledge of the pathophysiology of SARS-CoV-2 and how it relates to neurological disorders like stroke can aid in the development of novel treatment strategies. It is important to evaluate the data obtained from randomized controlled trials (RCTs) of COVID-19 vaccines and assess their long-term safety, which can shed some light on whether the vaccines can predispose to SD or SUD. Vaccine composition and different COVID-19 vaccine types must be evaluated for their role in the pathogenesis of SD and SUD. Given the exorbitant prevalence of co-morbidities, including uncontrolled diabetes and hypertension, chronic alcoholism, and other debilitating diseases, it is difficult to attribute previous SARS-CoV-2 infection and COVID-19 vaccination directly to the development of SD and SUD. A significant gray area exists since COVID-19 vaccinations are associated with a faster onset of thrombosis and atherosclerosis, or at the absolute least, adverse events, including vascular problems. Therefore, excluding vaccinations as a possible risk factor by using models may have negative effects on subsequent evaluations. Given the availability of data observed in other studies, it is important to exercise caution when ruling out vaccination’s association with SD and SUD. It is critical to comprehend the current surge in SD and SUD cases. Given the significant number of quality-adjusted life years at risk, long-term RCTs, prospective cohort studies, and heightened ecological monitoring for indications of an upward trend in SD and SUD are essential for a detailed understanding. In investigations of this kind, a multidisciplinary strategy comprising investigators, pathologists, geneticists, and forensic specialists, as well as prospective cohort studies, would improve the validity of the results. Exonerating the COVID-19 vaccinations would be prudent till then.

Considering the intricacy of post-COVID-19 health outcomes and vaccine-related problems, there is an increasing need to move toward precision medicine techniques. Proteomics, metabolomics, microbiomics, genomics, artificial intelligence (AI), and machine learning (ML) are cutting-edge therapeutic approaches used in precision medicine that are proposed for prediction, diagnosis, and treatment [68-73]. Furthermore, these could help identify those who are more likely to have certain unfavorable outcomes, such as unexpected or unexplained death [74]. Because AI-assisted monitoring systems can identify any early indications of serious or uncommon but potentially harmful consequences, they can advance pharmaceutical science and drug safety. These real-time technologies can be used to inform public health policy, detect risk effects, and enable tailored treatment approaches in clinical practice and vaccination safety surveillance [75]. Precision medicine is important because it allows for a more tailored and intimate understanding of the host-pathogen interaction, immune responses, and genetic vulnerability that may contribute to the negative effects following vaccination or infection [76].

Although there is a debatable linkage between COVID-19 immunization and SD, the public became anxious due to the growing reports of a potential relationship between the two [77]. Many people have been fearful and more hesitant about getting vaccines as a result of social media and inconsistent reporting from authorities. Therefore, it is imperative to ensure public health communication plays a significant role in easing these problems and informing people about the difficulties of vaccination compared to its advantages. To establish complete openness and preserve public confidence, healthcare experts, media teams, and social leaders should strive for timely updates and easily comprehensible scientific material that refutes vaccine myths with facts [78,79].

## Conclusions

Cytokine storm and undesirable inflammation resulting from the immune response, as well as exaggerated pro-inflammatory cytokine release that goes out of control following SARS-CoV-2 infection, could be a possible cause for SD. Of the recent post-COVID context, this may have also contributed to the rise in SD. Although SDs following immunization are concerning, it is critical to distinguish between cause and association. A thorough epidemiological investigation must consider the influence of underlying medical disorders, lifestyle choices, and even COVID-19 aftereffects. Compared to non-vaccinated individuals, vaccinated individuals may experience unfavorable effects due to the chemical components of vaccinations. This might have resulted in metabolic problems after vaccination as well as severe CVS and CNS symptoms. Some vaccines' mechanisms of action may involve unwanted immune system activation that isn't appropriate for a portion of the vaccinated population. The development of neurological and cardiovascular problems after immunization emphasizes the necessity for ongoing monitoring rather than diminishing the value of vaccinations. In the end, the data analyzed through this review demonstrates the need for a balanced view of COVID-19, PCFHS, and long-COVID, along with assessing, analyzing, and recognizing their long-term health affectation. Besides, COVID-19 immunization and its long-term health consequences require additional scientific validation and careful public health monitoring. Research on the role of microbial infections and their connection to SD has to be intensified in the age of emerging, remerging, and novel microorganisms.
